# Dental implants in patients treated with antiresorptive medication – a systematic literature review

**DOI:** 10.1186/s40729-016-0041-7

**Published:** 2016-04-04

**Authors:** Christian Walter, Bilal Al-Nawas, Tim Wolff, Eik Schiegnitz, Knut A. Grötz

**Affiliations:** 1 Department of Oral and Maxillofacial Surgery – Plastic Surgery of the Johannes Gutenberg-University Mainz, Augustusplatz 2, 55131 Mainz, Germany; 2 Department of Oral and Maxillofacial Surgery of the Dr. Horst Schmidt Clinic, Ludwig-Erhard-Str. 100, 65199 Wiesbaden, Germany

**Keywords:** Bisphosphonate associated osteonecrosis of the jaws, Bisphosphonate, Dental implant, Denture, Augmentation, Sinus lift, Antibiotics, Quality of life

## Abstract

**Objective:**

Bisphosphonate-associated osteonecrosis of the jaws (BP-ONJ) is triggered by inflammatory processes. Typical trigger factors are periodontal disease, denture pressure sores, and surgical interventions such as tooth extractions. Unfortunately there is only little data on how to proceed with implant therapy in patients with bisphosphonate treatment. This topic is not addressed in the German guidelines on medication-associated osteonecrosis. Therefore a systematic literature review was performed.

**Methods:**

The PICO design was used: (Patients) For which subclientel of patients with antiresorptive therapy (intervention) do dental implants have a benefit (control) compared to forgoing dental implants (outcome) in regards to oral rehabilitation and quality of life without having a substantial risk of BP-ONJ development? A PubMed search was performed including all studies dealing with this topic. Case reports and studies with less than 5 cases were excluded.

**Results:**

There is only very little data available, mostly retrospective case series. 50 articles were analyzed in detail. BP-ONJ can be triggered by dental implants and by dentures in patients with benign and malignant primary diseases. In most studies, analyzing osteoporosis patients only, no cases of BP-ONJ were observed in patients with implant therapy in the time span observed. There are no studies about implant therapy in patients with malignant diseases. Many case series analyzing the trigger factors for BP-ONJ describe dentures as one of the main causes. Perioperative antimicrobial prophylaxis has a benefit in the prevention of BP-ONJ development.

**Conclusion:**

Successful implant therapy is possible in patients receiving antiresorptive therapy. The possibility of osteonecrosis development needs to be explained to the patient. An individual risk assessment is essential, taking the primary disease with the medication and further wound-healing-compromising diseases and medications into account. If possible, bone augmentations should be avoided, and a perioperative antimicrobiological prophylaxis is strongly recommended in these patients.

## Introduction

Bisphosphonate-associated osteonecrosis of the jaws (BP-ONJ) is a well-known side effect in patients receiving bisphosphonates (BP) due to e.g. osteoporosis, multiple myeloma or malignant diseases with metastases to the bone; prevalences range between 0.1% for patients with primary osteoporosis to 1% in patients with secondary osteoporosis and up to about 20% for special high risk subpopulations of patients with a malignant disease and further predisposing factors [1, 2]. In addition to general risk factors such as the primary disease implicating the antiresorptive therapy, the antiresorptive therapy itself, concomitant diseases and medications and other influencing systemic factors usually a further factor triggering the development of BP-ONJ can be identified such as periodontal disease, extractions, denture pressure sores, or implant insertion [3, 4]. Usually BP-ONJ occurs in patients of higher ages (69 years ±10 years [3]) due to the primary disease causing the BP therapy so that it is not unusual that these patients seek the dentist for oral rehabilitation where implant therapy and bone augmentation for optimal implant positioning might be considered to substitute lost teeth.

There are guidelines describing BP treatment as a contraindication for implant therapy in patients with an oncologic primary disease [5, 6] that say implant insertion should be avoided [7, 8]. On the other hand, there are studies describing the safety of dental implant surgery in patients with oral BP and osteoporosis with no occurring BP-ONJ cases [5]. However, there are cases of successful implant insertion in patients with malignant primary diseases and cases of BP-ONJ in patients with osteoporosis [9, 10]. Reviews mention that there are only very few retrospective studies with moderate strength of evidence addressing this topic [11] so that no final recommendation can be given [12]. Oral and intravenous BP are not seen as absolute contraindications for dental implant therapy and that dental implants can osseointegrate successfully. It is recommended to do a risk assessment and to inform the patient about the potential risk of BP-ONJ development [11–13].

A similar scenario is well known in patients with radiation of the jaws. Initially, radiation therapy was seen as a contraindication for implant insertion [14] because of osteoradionecrosis. In Germany meanwhile, implants are covered by the health insurance by law in some of these patients (§28 SGB V Sozialgesetzbuch). Due to xerostomia sufficient fixation of a denture is rather complicated, and implants can improve the situation and might reduce the incidence of osteoradionecrosis by avoiding pressure denture sores that could result in exposed bone and eventually osteoradionecrosis.

This development could be transferred to patients with antiresorptive treatment (bisphosphonates, denosumab) since implants might reduce the incidence of BP-ONJ due to the lack of denture pressure sores in these patients. As well, denture pressure sores have been described by many authors as the triggering factor for BP-ONJ [15].

The German guidelines on bisphosphonate- and medication-associated osteonecrosis of the jaws state that there might be a limitation in the indication of implant insertion in these patients, but the implant-based oral rehabilitation was not a part of these guidelines [1]. To address this deficit in the literature, the rationale of this literature review was to find out which patients with antiresorptive therapy (BP, denosumab) benefit from dental implants without being exposed to an unreasonable high risk of osteonecrosis development.

## Review

### Methods

A systematic review was performed in accordance with the preferred reporting items for systematic reviews and meta-analyses protocols (PRISMA-P),

#### Focused question

The review was performed using the PICO design.

- Patients: For which subclientel of patients with antiresorptive therapy
- Intervention: do dental implants have a benefit
- Control: compared to forgoing dental implants
- Outcome: in regards to oral rehabilitation and quality of life without having a substantial risk of BP-ONJ development.

#### Search strategy

In June 2015, a PubMed search was performed by TW looking for all available articles; no limitation on the publication date was imposed. The search was modified by CW, BA, ES and KAG so that 24 more articles were identified.

**Fig. 1:**
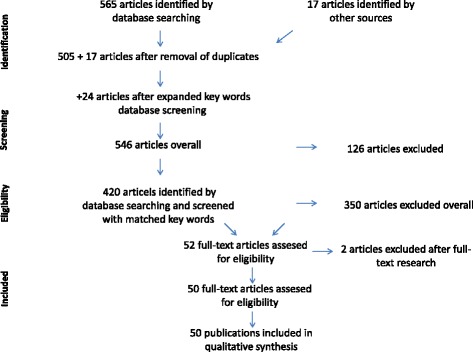
Flow diagram of literature search and selection

To address the topics, the search terms bisphosphonate and denosumab were used in combination with the following search terms: osteonecrosis, jaw, dental implants, periimplantitis, denture, augmentation, sinus lift, antibiotics, xerostomia, CTX, medication time, masticatory efficiency, tmj disorder, prevention, oral health related quality of life [NOT oral cavity cancer], prognosis dental implant, persisting alveolar socket, sharp bone edges [NOT children NOT osteogenesis imperfecta, ossification [NOT children NOT osteogenesis imperfecta], bone remodeling, and post extraction [NOT children NOT osteogenesis imperfecta]. 17 articles were added due to the manual search (Table 1 and Fig. 1).

**Table 1:** PubMed literature search with the total number of hits for each topic and the number of included articles for each topic. For each topic the search terms (ST) are given

Topic	Identified (*n*)	Included (*n*)
Outcome osteonecrosis risk. ST (bisphosphonate OR denosumab) AND osteonecrosis jaw AND		
Dental implant OR periimplantitis	105	18
Denture	49	12
Jaw augmentation OR sinus lift	7	0
Antibiotics AND dental implant	16	0
Xerostomia	4	0
CTX	35	0
Medication time	16	0
Outcome mastification. ST		
Masticatory efficiency AND dental implant	61	0
TMJ disorder AND dental implant AND prevention	10	0
Outcome quality of life. ST (bisphosphonate OR denosumab) AND		
Quality of life AND dental implant	5	1
Oral health related quality of life AND dental implant	1	0
Outcome prognosis remaining dentition. ST (bisphosphonate OR denosumab) AND		
Prognosis remaining dentition AND dental implant	0	0
Outcome prognosis future implants. ST (bisphosphonate OR denosumab) AND		
Dental implant AND prognosis	16	0
Persisting alveolar socket	2	2
Sharp bone edges AND jaw NOT children NOT osteogenesis imperfecta	2	0
Ossification AND jaw NOT children NOT osteogenesis imperfecta	66	0
Bone remodeling AND post extraction AND jaw NOT children NOT osteogenesis imperfecta	7	
Radiologic changes AND jaw NOT children NOT ostegenesis imperfect AND dental implant	1	0
Hand search		17
Total	403	50

#### Study inclusion and exclusion criteria

Inclusion criteria were:

Prospective (randomized controlled, non-randomized controlled, cohort) and retrospective studies (controlled, case control, single cohort) and case series dealing with dental implants in patients with antiresorptive therapy. Studies that had less than five patients or cases were excluded as well as studies whose cases lacked data or were not clearly defined. The studies had to be published in either English or German.

Studies that did not meet the inclusion criteria were excluded.

In the first step, the titles and abstracts were screened for eligibility. In the second step, all full-articles were evaluated.

#### Quality assessment of selected studies

Due to the available data, an explicit quality assessment was not performed.

#### Data extraction and method of analysis

A table was generated and used to collect the relevant information.

## Results

Out of 606 articles 556 articles were excluded because they were either duplicates, case reports, narrative reviews, case series with less than 5 cases or were not associated with the topic at all (Table 1 and Fig. 1). Some of the articles analyzed more than one outcome and are referred to several times. Since the available literature is very inhomogeneous with a low level of evidence a statistical analysis was not performed and the following results are descriptive only.

### Dental implants/periimplantitis

The literature dealing with this topic can roughly be separated into three groups:

- BP-ONJ case series exclusively triggered by implants in patients with malignant and benign diseases [9, 10, 16–19],
- BP-ONJ case series analyzing case series of BP-ONJ of which varying amounts are caused by implants in part among patients with malignant and benign diseases [20–23] and
- implant studies performed exclusively in patients with benign diseases, mostly osteoporosis [24–31]. In very few of these studies, the primary disease was not given, but the prescribed bisphosphonates strongly suggest osteoporosis as the primary disease (see Table 2).

**Table 2:** Included literature

Implant										
Author Year Reference	Study type	Patients	Primary disease in BP patients (n)	BP BP-Th (years min- max)	BP-ONJ cases due to implants	Implants (n)	Implant insertion in all patients (patients [n]) Point of time			Comment
							before BP therapy	during BP therapy	after BP therapy	
Al-Sabbagh 2015 [24]	RS CS	203 patients with 515 implants; 20 out of those patients with osteoporosis and oral BP	Osteoporosis 20	Oral BP > 3	0	46	n.s.	n.s.	n.s.	All patients with implant therapy from 08/2000 until 05/2004 were contacted and data was collected by interview (in person/per telephone). 203 patients with 515 implants; in 20 patients (46 implants) with osteoporosis and oral BP no ONJ occurred no implant was lost. There is no data regarding the implant success in the patients without osteoporosis.
Nisi 2015 [20]	RS CS	90 patients with established ONJ some of them with implants	Malignoma 90	Z n.s.	9	n.s.	n.s.	n.s.	n.s.	All patients with BP-ONJ from 01/2004 until 12/2015 were retrospectively analyzed. 78% had an additional radiation. It is not clear if the head and neck region was affected and if the implant patients were affected. The study describes the cumulative BP dose, smoking, steroid intake and the maxillary location as risk factors for an increases BP-ONJ stage.
Holzinger 2014 [9]	RS CS	13 patients with established ONJ due to dental implants	Osteoporosis 5 breast cancer 3 lung cancer 1 Langerhans cell histiocytosis 1	Z A P I- 0.5 – 9 0 – 15.6	13	47	3	7	3	All patients with BP-ONJ from 04/2004 – 07/2012 were analyzed. Among those were 13 patients (47 implants) with implants as a trigger. 30 implants had to be removed. It takes longer for BP-ONJ development if implants are placed during or after BP treatment compared to implants being inserted before BP treatment.
Lopez-Cedrun 2013 [10]	RS CS	9 Patients with established ONJ due to dental implants	Osteoporosis Polymyalgia rheumatica	A I R 0.5 - 10	9	57	-	9	-	Retrospective multicenter study analyzing all patients with BP-ONJ due to dental implants from 01/2009 – 06/2012. The authors state that the ONJ was more common in the mandible (8/9) and more often in the premolar and molar region. 28 implants maxilla → 1 BP-ONJ 29 implants mandible →8 BP-ONJ
Tam 2013 [16]	RS CS	6 patients with established BP-ONJ due to dental implants	Osteoporosis 4 breast cancer 1 multiple myeloma 1	A Z 1.5-6	6		-	6	-	All patients with BP-ONJ due to dental implants from 2009 – 2011 were analyzed; 3 patients with BP-ONJ in the posterior maxilla 3 patients with BP-ONJ in the mandible (2 distal, 1 anterior)
Kwon 2014 [17]	RS CS	19 patients with established BP-ONJ due to dental implants	Osteoporosis 18 multiple myeloma 1	A I P R Z 1 - 5	19	n.s.	3	16	-	All patients with BP-ONJ due to dental implants from 06/2008 – 12/2011 were analyzed. 8 patients with BP-ONJ in the maxilla, 9 patients with BP-ONJ in the mandible, 2 patients with BP-ONJ in mandible and maxilla
Jacobsen 2013 [18]	RS CS	14 patients with established BP-ONJ due to dental implants	Osteoporosis 5 Breast cancer 5 multiple myeloma 2 prostate cancer 1 lung cancer 1	A I P Z Average BP duration 3.2 osteoporosis; 4.2 malignant disease	14	n.s.	n.s.	n.s.	n.s.	The authors state that implants placed posterior are of higher risk than implants in the anterior region. 4 patients had implants in the posterior maxilla, 5 in the posterior mandible and 3 in the anterior mandible. In one patient implants were removed and new implants were inserted at the same site with continuing problems. In one patient a sinus lift was performed
Famili 2011 [25]	RS CS	211 female patients with 592 dental implants, out of those 120 older than 50 y with 347 implants out of those 22 with BP and 75 implants	Osteoporosis 21 osteoarthritis 1	A I <1 - > 5	0	75	n.s.	At least 20	n.s.	All female patients with implant therapy from 01/2008 – 06/2010 were analyzed. Among those 22 with oral BP therapy. One implant did not heal and was successfully replaced
Kwon 2011 [21]	RS CCS but not focused on dental implants → RS CS	Biochemical bone markers were evaluated in 23 osteoporosis patients with established BP-ONJ	Osteoporosis	A 2.5 - 5	2	n.s.	n.s.	n.s.	n.s.	It is not clear, when and how the 23 BP-ONJ patients were recruited. 61 BP control patients. 2 patients developed BP-ONJ due to implants CTX was evaluated at the time of ONJ diagnosis and not at the time point of any possible BP-ONJ triggering intervention.
Koka 2010 [22]	RS CS	370 patients over 50 years old with 818 implants. 233 patients could not be reached so that the phone interview was conducted with remaining 137 patients: 55 BP patients and 82 non-BP patients	Osteoporosis	A & n.s. <3 - > 5	0	121	-	55	-	All patients from 11/2006 – 05/2009 that had not denied access to data for research purposes. None of the BP patients had a drug holiday. 121 implants were inserted, one did not survive. The patients were not examined only a phone interview was conducted. The control group consisted of 82 non-BP users with 166 implants (163 survived, 2 losses in 1 patient). 233 patients could not be reached by phone and were excluded.
Lazarovici 2010 [19]	RS CS	27 patients with established ONJ due to dental implants	Osteoporosis 11 multiple myeloma 7 breast cancer 7 prostate cancer 2	A P Z average BP duration A 5.7 Z 1.4 P 4.2	27	n.s.	2	25	-	All patients from 04/2003 – 01/2009 with BP-ONJ and dental implants. 15 patients had implants in the posterior mandible, 5 in the anterior mandible, 4 in the posterior maxilla, 3 in the anterior maxilla
Goss 2010 [26]	RS CS	Questionnaire to 46 dentists placing either > 50 implants/y or treat BP-ONJ in South Australia	Osteoporosis in the 7 patients with implant loss	A R in the 7 patients with implant loss	7	≥9	4	3	-	46 dentists placed approximately 28,000 implants in 16,000 patients. There is no number given how many patients received BP. 7 implants were lost in patients with BP
Lo 2009 [27]	RS CS questionnaire	Questionnaire to 13,496 patients with oral BP therapy, 8,572 answered, 2,159 reported dental symptoms, 1005 were examined, 9 BP-ONJ	n.s.	A I R ≥1	1	n.s.	n.s.	n.s.	n.s.	13,946 questionnaires were sent, 5,374 did not participate, 9 ONJ were identified and 1 was associated with an implant loss and a tooth extraction. The bisphosphonates had been administered before implant insertion
Bell 2008 [28]	RS CS	42 patients with BP therapy and oral bone grafting or implant placement	n.s.	A R I	0	100 or 101 both numbers are given in the paper	-	42	-	All patients from ??/1990 - ??/???? (paper published in 2008) with BP treatment prior to implant therapy were analyzed. 5 implants failed, no patient with more the 1 implant loss, all implants successfully replaced. 30 patients received an additional bone augmentation (socket graft, sinus lift, closed sinus lift, guided tissue regeneration, or tunnel graft).
Grant 2008 [29]	RS CS	Questionnaire to all 1,319 female patients over 40 y and with implants, 458 patients responded, 115 out of those had oral BP, 72 patients came to a follow-up	n.s.	A I R Mean 3.2	0	456 in the 115 patients	26 out of the 115	89 out of the 115	-	All 1,319 patients over 40 y of age with implant therapy between 01/1998 – 12/2006 were contacted.
Fugazotto 2007 [30]	RS CS	61 patients out of two private practices with oral BP	n.s.	A R Mean 3.3	0	169	-	61	-	All 61 patients with oral bisphosphonates with implant therapy between 01/2005 – 12/2005 were analyzed. 43 immediate implants 1 Pat had exposed bone at a different location that was treated by debridement. At the next control there was granulated soft tissue.
Jeffcoat 2006 [31]	PS	50 patients with 210 implants 25 patients with oral BP 102 implants 25 patients without BP 108 implants	Osteoporosis 25	A R 1 - 4	0	102	-	102	-	Longitudinal single-blind controlled study Two-stage osseointegrated implants in all patients, no BP-ONJ
Marx 2005 [23]	RS CS	119 patients with BP-ONJ	n.s. for the patients with implants	n.s. for the patients with implants, in 1 case Z & P	4	n.s.	n.s.	n.s.	n.s.	RS with 119 ONJ patients, 4 due to dental implants
Denture										
Author Year Reference		Study type	Patients	Primary Disease in BP-ONJ		BP/Denosumab		BP-ONJ Cases	Comment	
Nibbe 2015 [15]		RS	424 patients with oral/IV BP or denosumab. 128 patients with IV BP or denosumab - further investigation of this group 68 patients with dentures	n.s. in all cases		Oral/IV BP Denosumab		16	424 patients with oral or IV BP were analyzed, 21 BP-ONJ, only IV BP patients for further analysis 34 removable dentures → 11 BP-ONJ 34 fixed partial denture → 5 BP-ONJ 60 patients without denture → 5 BP-ONJ ONJ only in patients with IV BP or denosumab	
Hasegawa 2012 [43]		RS	Questionnaire was sent to 248 medical institutions regarding BP-ONJ 250 patients 99 with dentures 151 without dentures	n.s.		Oral/IV BP		99	151 osteonecrosis patients without denture had a longer osteonecrosis free time. Most ONJ in the mandible with a focus on the premolar and molar region 154 patients with IV BP, 102 with oral BP, 7 both	
Jabbour 2012 [35]		RS	14 patients with BP-ONJ 4 due to dentures	Osteoporosis 2 kidney cancer 1 breast cancer 1		A P		4	RS analyzing the reason for osteonecroses	
Vahtsevanos 2009 [39]		RS	1,621 patients with IV BP	n.s.		n.s. for the denture patients		24	24 out of 80 BP-ONJ patients denture as triggering factor diseases and BP for the patients with dentures n.s. I P Z were used as BP in the BP-ONJ patients.	
Kos 2010 [36]		RS	34 patients with BP-ONJ	n.s.		n.s. for denture patients		3	34 patients with osteonecrosis. A I P R Z were used as BP it is not clear what the patients with the dentures received and which primary disease was present. BP-therapy for all patients 0.3 – 8 y	
Carmagnola 2008 [34]		RS	39 oncologic patients with BP	Multiple myeloma 2 breast cancer 3 prostate cancer 1 kidney cancer 1		P Z		7	7 out of 20 BP-ONJ patients had an osteonecrosis due to denture pressure sores BP given for 1.1 – 6.8 y	
Walter 2008 [40]		CSS	43 patients with prostate cancer out of those 21 patients with denture out of those 6 with ONJ	Prostate cancer		Z		1	1 denture induced ONJ	
Kyrigidis 2008 [38]		CCS	20 breast cancer patients 40 matched controls	Breast Cancer		Z		8	20 patients with breast cancer and osteonecrosis, 8 with dentures use of dentures associated with BP-ONJ	
Kumar 2008 [37]		RS CS	13 patients with BP-ONJ	Osteoporosis 4 breast cancer 1 multiple myeloma 1		A Z		6	6 out of 13 patients denture as the trigger factor	
Yarom 2007 [42]		RS CS	11 patients with BP-ONJ	Osteoporosis		A		2	2 out of 11 BP-ONJ triggered by denture Alendorante was given for 2 and 6 y 2 patients (1 osteoporosis, 1 rheumtoid arthritis) had an implant related BP-ONJ in the posterior mandible, BP was given for 5 and 7 years	
Walter 2007 [41]		RS CS	163 patients with an osteonecrosis, 17 BP-ONJ	Multiple Myeloma		P		1	1 BP-ONJ due to a denture pressure sore in the mandible P was given for 5 y	
Bamias 2005 [33]		PS	252 patients with BP 17 with BP-ONJ	Multiple Myeloma		n.s.		2	2 BP-ONJ due to a denture pressure sore I P Z for all 252 patients	
Quality of life										
Author Year Reference		Patients				Comment				
DeBaz 2015 [44]		524 patients asked to fill out the survey 237 completed survey 3 groups: 64 dental implant supported prosthesis 47 non-implant supported fixed restoration 60 non-implant supported removable restoration 66 no restoration of missing teeth				The quality of life assessment: occupational score health score emotional score sexual score The patients dental implant supported prosthesis had the overall best score regarding quality of life compard to the other groups In total 134 patients reported oral BP, 51 IV BP, 10 patients denosumab In the implant group 35 patients received oral BP, 12 IV BP, 3 denosumab no ONJ				
Persisting alveolar socket										
Author Year Reference		Study type	Patients			Comment				
Hutchinson 2010 [46]		CSS	10 patients with stage 0 BP-ONJ			Consistent findings of regional or diffuse osteosclerosis, density confluence of cortical and cancellous bone, prominence of the inferior alveolar nerve canal, thickened sclerotic lamina dura, periradicular radiolucencies, cortical disruption, and persisting alveolar sockets.				
Grötz 2006 [45]		RS CS	42 patients with BP-ONJ			Consistent findings of persisting alveolar sockets.				
Hand search										
Author Year Reference		Study type	Patients			Comment				
Grötz 2012 [1]		Guideline				German guidelines on bisphosphonate-associated osteonecrosis of the jaws (BP-ONJ) and other medication-related necroses of the jaw				
Grötz [52]						Description on many important aspects on implant surgery in bisphosphonate patients or patients with other resorptive medications.				
Grötz 2013 [53]		Review				The authors state the necessity for an individual risk assessment. The evaluation of dentures vs. implants. It is suggested to not place immediate implants in patients with antiresorptive therapy, atraumatic surgery with perioperative antibiotics, the necessity of a recall and the avoidance of bone augmentations				
Grötz 2010 [54]		Review				The authors provide an algorithm how to proceed with patients receiving BP seeking implant therapy. The authors state the necessity for an individual risk assessment and avoidance of bone augmentations				
Krimmel 2014 [55]		RS	50 patients with BP-ONJ			DMFT of all patients 20.5 ± 4.2 disease free interval for patients with DMFT < 20: 39.7 ± 1.1 months disease free interval for patients with DMFT > 20: 14.4 ± 2.8 months The DMFT had no influence on the overall healing rate of BP-ONJ				
Tsao 2013 [56]		CCS	63 patients 22 BP-ONJ patients 41 matched controls			Caries similar between groups Periodontal disease associated with BP-ONJ (pocket depth, IgG serum titer against Porphyromonas gingivalis, IL 1ß level in gingival cervical fluid)				
Thumbigere-Math 2013 [57]		CCS	73 patients 25 BP-ONJ patients 48 matched controls			BP infusions BP-ONJ 38.4 and control 18.8 BP-ONJ vs control: missing teeth: 7.8 vs 3.1 clinical attachment level: 2.18 vs 1.56 radiogic bone loss at teeth > 50%: 20% vs. 6%				
Martin 2010 [58]		CSS	8,752 patients with oral BP returned dental survey (62% response rate) 589 patients with dental implants			16 patients with 26 implant failures 8 patients with failure of 12 implants in the maxilla 9 had failure with 14 implants in the mandible				
Shabestari 2009 [59]		RS	21 female osteoporotic women with oral BP and 46 implants			No BP-ONJ, no signs of peri-implantitis				
Zahid 2011 [60]		RS	362 patients with implants 26 BP patients with 51 implants			3 implants failed Patients with BP had more thread exposure				
Memon 2012 [61]		RS	200 patients BP: 100 women with 153 implants control: 100 women with 132 implants			Success equal for both groups 93.5 (BP) vs. 95.5 (control) crestal bone change from implant insertion to stage two surgery: no difference between the groups				
Yip 2012 [62]		CCS	337 patients 114 patients with implant failure 223 patients without implant failure			% of patients using BP Implant failure group: 9.65% no implant failure: 4.04				
Walter 2014 [3]		RS	504 patients with osteonecrosis 227 with BP-ONJ			7 out of 227 patients with BP-ONJ implant as trigger factor				
Lopez-Jornet 2011 [63]		Animal study	120 rats with pamidronate treatment and molar extraction 60 with penicillin 60 without penicillin			Osteonecrosis rate Penicillin group: 18 → 34.6 % no penicillin group: 5 → 9.61%				
Montefusco 2008 [51]		RS	178 patients with multiple myeloma und BP treatment 75 patients with dental procedures 32 with antibiotics 43 without antibiotics			ONJ rate with antibiotics: 0 without antibiotics: 8 Antibiotic prophylaxis can reduce the incidence of BP-ONJ				
Kyrgidis 2012 [50]		PS	Group 1: BP-ONJ breast cancer 21 Group 2: breast cancer 21 Group 3: oral cancer 22			Quality of life assessment before surgery Group 1 is affected in many factors such as pain, swallowing, senses, social eating, social contacts				
Boquete-Catro 2015 [32]		Review	Analysis of patients with denosumab associated ONJ			No implant related ONJ reported				

A: Alendronate; CCS: Case control study, CS: Case series; CSS: Cross sectional study; D: Denosumab; I: Ibandronate; LSBCD: Longitudinal single-blind controlled study; n.s.: Not specified; P: Pamidronate; PS: Prospective Study; R: Risedronate; RS: Retrospective study, Z: Zoledronate

In the BP-ONJ case series (a) and (b), the distribution of BP-ONJ patients between malignant and benign diseases is more or less even, e.g. Holzinger describes 13 patients: 5 osteoporosis, 3 breast cancer, 3 multiple myeloma, 1 lung cancer and 1 Langerhans cell histiocytosis patient [9]. Lopez-Cedrun exclusively found patients with benign diseases: 8 osteoporosis and 1 polymyalgia rheumatic. Jacobsen found 14 patients: 5 osteoporosis, 5 breast cancer, 2 multiple myeloma, 1 prostate and 1 lung cancer patient.

However, implant studies (c) were exclusively performed in patients with benign diseases. Nearly all of these studies do not report a single BP-ONJ triggered by the implant insertion.

A systematic review analyzing the sparse literature on clinical denosumab trials mentioning osteonecroses did not describe dental implants as a trigger [32].

There is no literature describing periimplantitis in these patients.

### Denture

The literature dealing with this topic can be separated into two groups:

- BP-ONJ case series for which varying amounts are caused by dentures in patients with malignant and benign diseases [2, 33–42] and
- studies performed on BP patients with dentures analyzing the frequency of BP-ONJ [15, 43].

Here as well, no imbalance regarding the dignity of the primary disease could be found. Jabbour describes 2 osteoporosis, 1 kidney and 1 breast cancer patient [35]. Kumar found 4 osteoporosis patients, 1 breast cancer patient and 1 patient with multiple myeloma.

Nibbe [15] analyzed 128 patients with IV BP or denosumab separated into 3 groups. In the first group 5 out of 60 patients with no denture had an osteonecrosis (8%), in the 2nd group 5 out of 34 patients with a fixed partial denture had an osteonecrosis 15%, and in the 3rd group 11 out of 34 patients with a removable denture had an osteonecrosis (32%). Kyrgidis determined that dentures increase the risk of BP-ONJ development [38].

### Augmentation/sinus lift/antibiotics/xerostomia/CTX

There was no literature available meeting the inclusion criteria. There is evidence in the literature that sinus lifts can be successful [28] and might contribute to BP-ONJ development [18].

### Outcome masticatory efficiency/TMJ disorder

There was no literature available meeting the inclusion criteria.

### Outcome quality of life (QoL)

There is nearly no literature available on the change of the quality of life due to implants in patients with antiresorptive therapy. One article could be identified [44] that analyzed the quality of life in partially edentulous osteoporosis patients that were restored with (1) a dental implant-supported prosthesis, (2) a non-implant-supported fixed restoration, (3) a non-implant-supported removable restauration, and (4) no restoration that showed a statistically significant difference regarding the improvement of the QoL in patients with a dental implant-supported prosthesis compared to the other sub-groups. Out of the 237 patients, 134 patients had an oral BP and 51 patients an IV BP therapy [44].

### Outcome remaining dentition

No articles could be found regarding the prognosis of the remaining dentition depending on implant therapy, neither for patients with bisphosphonate nor denosumab treatment.

### Outcome future implants

There are no reliable parameters indicating the success of implants in patients with anti-resorptive therapy. The risk of osteonecrosis development has already been described in the chapter referring to the osteonecrosis risk. Two articles mentioned the radiologic finding of a persisting alveolar socket as a marker indicating the osteonecrosis risk [45, 46]. In addition, other radiologic changes in patients with bisphosphonates have been described, such as regional or diffuse osteosclerosis, confluence of cortical and cancellous bone, prominence of the canal of the inferior alveolar nerve, a prominent lamina dura, radiolucences around the apex and cortical disruptions [46]. Their existence reflects the changes in the bone remodeling due to the anti-resorptive therapy and might be associated with a higher osteonecrosis risk, but there is no evidence supporting this theory.

## Discussion

Even latest guidelines and statements dealing with medication associated osteonecrosis of the jaws such as the American [7, 8], Scottish [47], Swiss [6] or German [1] do not address implant therapy in these patients in detail. Due to this lack of data a systematic literature review was performed to fill this gap. Unfortunately the literature dealing with this topic is very sparse and consists mainly of case reports, case series, and a few retrospective studies. Regarding the topics augmentation, masticatory efficiency, TMJ and the impact on the remaining dentition no literature met the inclusion criteria or no literature was available at all.

Surgical procedures such as implant insertion and potential complications such as periimplantitis are associated with an inflammatory condition and can potentially trigger a BP-ONJ. The risk of developing BP-ONJ is higher the more potent the BP are and the longer they were administered [1]. There is lots of literature supporting dental implant therapy in patients with antiresorptive medication in benign primary diseases, with only a few patients developing BP-ONJ (Table. 2). In contrast, the literature regarding successful implant therapy in malignoma patients is very sparse. The majority of publications on malignoma patients describes scenarios with BP-ONJ development (Table 2).

In many cases a denture would be the alternative treatment option to dental implants but many BP-ONJ cases in patients with benign and malignant diseases are caused by denture pressure sores (Table 2), so when deciding whether a patient is eligible for implant therapy, this fact should be considered as well. The consideration needs to take into account how much the patient might benefit from the implant itself, the risk of causing an osteonecrosis due to the procedure and the likelihood of preventing an osteonecrosis by avoiding dentures and denture pressure sores.

In the consideration of a potential implant insertion the need for a bone augmentation or a sinus lift needs to be considered as well. Although there are only very few cases in the literature with augmentation of bone/sinus lift [18, 28], these procedures are linked to a functioning vascular recipient site with working osteoclastic resorption and osteoblastic bone formation, and this is compromised in patients with antiresorptive therapy. Due to the denudation of the bone at the recipient site the vascular situation might be even more compromised, possibly resulting in more BP-ONJ cases so that any kind of augmentation should be considered with extreme care.

Dental implants can improve the Qol in patients with antiresorptive therapy (bisphosphonate/denosumab) [44] analogous to patients without antiresorptive therapy [48]. A recently performed systematic review on masticatory performance, bite force, nutritional state and patient’s satisfaction showed that implant-supported dentures were associated with high patient satisfaction regarding denture comfort and bite force. But interestingly these outcomes were not always related with an increase in general QoL [49]. There is no reason, why this should be any different in patients with antiresorptive therapy in the event of implant success. On the other hand the occurrence of BP-ONJ has a huge impact on the QoL of affected patients since the patients report higher negative affection by pain, problems swallowing and social eating even compared to patients with head and neck squamous cell carcinoma [50]. Therefore prevention of BP-ONJ should be one of the primary aims.

The risk of triggering a BP-ONJ by implant therapy in patients with benign diseases seems to be rather small. The risk in patients with malignant diseases is hard to describe since mostly negative examples are published but not the total number of patients receiving implant therapy. The distribution clearly illustrates the necessity for an individual risk assessment as it is recommended by most guidelines and the importance of explaining the possibility of BP-ONJ development to the patient. The individual risk is influenced by the primary disease and its treatment, such as the antiresorptive medication (substance, duration of application, frequency of application), concomitant therapy, further diseases (e.g. diabetes), further treatments (e.g. head and neck radiation), further factors (e.g. smoking) and existence of former osteonecrosis/present osteonecrosis. Next to this, the patients need to be compliant with an appropriate motivation for oral hygiene and the necessary skills to transfer this. Infectious foci should be treated before implant therapy to further reduce the risk of osteonecrosis development. The surgical sites should be followed up clinically (persisting sharp bone edges without any tendency to remodel) and radiologically (e.g. persisting alveolar sockets) to identify a compromised wound healing that might be associated with a higher BP-ONJ risk.

The use of bone markers is discussed controversially in the literature, and no clear recommendation can be given at the moment [1, 8]. In these cases, where an implant is planned, a perioperative antimicrobial prophylaxis should be administered, similar to the prophylaxis suggested in other surgical procedures performed in these patients, since this seems to be a tool to decrease BP-ONJ frequency [1, 51]. There is no literature on patients with antiresorptive medications other than bisphosphonates, and so the recommendation is to proceed with these patients similarly to patients on bisphosphonates.

Unfortunately literature with high evidence is rare. Dental implants are possible in patients with antiresorptive therapy but an individual risk assessment should be performed and alternative treatment options should be considered keeping the scenario of BP-ONJ development in mind. Implant survival and success rate alone are not sufficient to evaluate the implant supported rehabilitations in patients with bisphosphonates. Even more important is the risk of triggering an osteonecrosis in relation to the possible gain of QoL by an implant supported therapy.

## Conclusions

Successful implant therapy is feasible in patients receiving antiresorptive therapy. The risk of osteonecrosis development needs to be explained to the patient. An individual risk assessment is essential, taking the primary disease with the medication and further wound-healing-compromising diseases and medications into account. If possible, bone augmentations should be avoided, and a perioperative antimicrobiological prophylaxis is strongly recommended in these patients.

## Abbreviations

BP: bisphosphonate
BP-ONJ: bisphosphonate-associated osteonecrosis of the jaws
CTX: C-terminal telopeptide of the type I collagen
PICO: patients intervention control outcome
PRISMA-P: preferred reporting items for systematic reviews and meta-analyses protocols
QoL: quality of life
ST: search term
TMJ: temporomandibular joint
